# Advances in Freezing and Thawing Meat: From Physical Principles to Artificial Intelligence

**DOI:** 10.3390/foods15020396

**Published:** 2026-01-22

**Authors:** Qianrui Xia, Shiwei Yan, Ming Huang, Kunjie Chen, Jichao Huang

**Affiliations:** 1 Department of Engineering, Nanjing Agricultural University, Nanjing 211800, China; 2024112019@edu.nju.cn (Q.X.); 2024812074@edu.nju.cn (S.Y.); kunjiechen@njau.edu.cn (K.C.); 2 Department of Food Science and Technology, Nanjing Agricultural University, Nanjing 211800, China; mhuang@njau.edu.cn

**Keywords:** meat, freezing and thawing technology, ice crystal morphology, artificial intelligence, numerical simulation

## Abstract

With the sustained expansion of global meat consumption, advanced freezing and thawing technologies have become essential to preserve quality and extend shelf life within the food supply chain. This review systematically consolidates recent progress by examining fundamental principles, conventional techniques, emerging multi-physics methods (e.g., high-pressure-, ultrasound-, and electric field-assisted processing), and the integration of artificial intelligence (AI). It details the mechanism of ice-crystal formation and its impact on meat quality attributes. While conventional methods remain prevalent, their limitations in controlling ice crystallization are evident. Emerging technologies demonstrate superior capability in regulating ice morphology, thereby mitigating cellular damage. AI applications, including numerical simulation, quality monitoring via machine learning, and predictive modeling of thawing parameters, show considerable potential to enhance processing efficiency—though challenges in data scarcity and model generalizability remain. Collectively, these advancements form an integrated “theory–technology–intelligence” framework, supporting the development of more sustainable, efficient, and quality-focused meat processing systems.

## 1. Introduction

Meat constitutes an essential component of human diets worldwide, serving as a crucial source of high-quality proteins, essential amino acids, and bioavailable micronutrients that are fundamental to human health and physiological development [1,2,3]. The sustained growth in global meat consumption has intensified the technical challenges associated with quality preservation throughout the supply chain, particularly in maintaining product safety and nutritional integrity during storage and distribution [4]. In this context, freezing and thawing technologies have emerged as indispensable processing methods for shelf-life extension while preserving key organoleptic and nutritional attributes.

However, these preservation methods induce complex physical and biochemical changes that often compromise meat quality. Ice crystal formation during freezing causes structural damage to cellular networks, while protein denaturation and lipid oxidation during thawing further degrade functional properties [5]. Conventional techniques, despite their economic advantages, face limitations including inefficient heat transfer, uncontrolled ice crystal growth, and substantial energy requirements [6].

Recent advances have introduced innovative approaches utilizing multi-physical field interactions. Techniques such as high-pressure freezing and radio frequency thawing achieve superior ice crystal control through enhanced nucleation and crystallization suppression [7,8,9,10,11]. However, industrial implementation encounters constraints, including equipment costs and optimization complexity [12,13]. The integration of artificial intelligence represents a transformative development, enabling precise prediction of crystal dynamics and autonomous parameter optimization [14,15]. Through the integration of intelligent sensor technology and advanced deep learning technology, it is expected to achieve real-time monitoring and intelligent analysis of the food industry chain [16].

This review systematically examines the technological evolution from fundamental mechanisms to intelligent systems, establishing a scientific foundation for advanced meat processing that meets global supply chain demands.

## 2. Progress in Basic Theory of Freezing and Thawing Technology

### 2.1. Ice Nucleation

**Figure 1:**
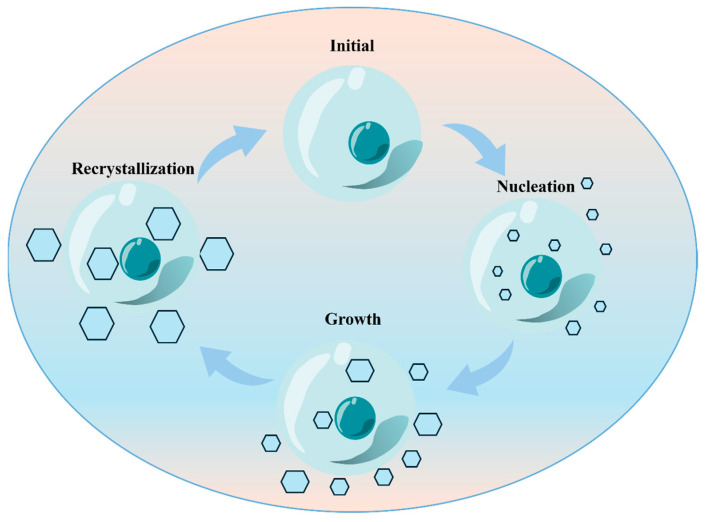
Ice crystal nucleation process.

Ice nucleation represents the critical stage at which supercooled water undergoes a phase transition into the solid state [14,17]. This initial process can proceed via two distinct pathways. The first is homogeneous nucleation, which occurs in pure water and requires profound supercooling for water molecules to spontaneously assemble into ice nuclei without any foreign surfaces [18]. The more prevalent mechanism is heterogeneous nucleation, wherein ubiquitous impurities—such as proteins, mineral particles, or other nucleating agents—act as templates for crystal formation. This pathway significantly reduces the energy barrier for nucleation, thereby initiating ice formation at considerably higher temperatures. The above is the complete process of ice crystal nucleation, which can be vividly described in Figure 1.

The nucleation rate *J* is exponentially dependent on the degree of supercooling [19]. Among them, $\Delta G^{*}$ specifically targets the critical radius $r^{*}$, which is the key energy threshold for the nucleation process. Current research focuses on controlling nucleation sites and rates to regulate ice crystal size and distribution.

(1) $$J=J_{0}\exp\left(-\frac{\Delta G^{*}}{k_{B}T}\right)$$

$J_{0}$: Pre-exponential factor;

$\Delta G^{*}$: Gibbs free energy difference of critical crystal nuclei;

$k_{B}$: Boltzmann constant;

T: Absolute temperature.

### 2.2. Crystal Growth: The Ice Builds Up

Once the first stable nuclei are established, the ice crystal growth stage begins—a phase of orderly expansion where water molecules from the surrounding liquid diligently attach themselves to the existing crystalline lattice. However, this growth is not without its consequences. Figure 1 depicts in detail the complete process of ice crystal nucleation.

The process releases latent heat, which warms the local environment and complicates the very supercooling that drove the initial nucleation. In this dynamic interplay, water molecules navigate to their designated spots on the crystal lattice, while solutes are selectively excluded, leading to the fascinating and diverse morphologies of ice crystals [20,21]. Managing this growth through rapid cooling is key to producing a fine, delicate ice crystal structure that is less damaging to meat’s cellular integrity.

### 2.3. Recrystallization: The Unwanted Makeover

During frozen storage, meat remains subject to quality changes. Recrystallization is a silent, often destructive process of microstructural rearrangement, driven primarily by inevitable temperature fluctuations. In a classic case of “the rich get richer,” smaller ice crystals dissolve (to reduce surface energy), and larger ones grow at their expense, a phenomenon known as Ostwald ripening [22].

As demonstrated in Table 1, this relentless coarsening of the ice crystal network is a primary culprit behind the quality degradation in frozen meats, leading to increased drip loss, textural deterioration, and flavor migration, Therefore, inhibiting recrystallization is the final, critical challenge in preserving the pristine quality of meat products throughout the cold chain.

## 3. Evaluation of Traditional Freezing and Thawing Technology

### 3.1. Evaluation of Traditional Freezing Technology

#### 3.1.1. Air Freezing

Air freezing refers to a method in which cold air is forcibly circulated during the freezing process and exchanges heat with the food surface through natural or forced convection [28]. As one of the most common freezing techniques, it is characterized by low cost and suitability for large-scale production. However, its relatively slow heat transfer rate often leads to the formation of large extracellular ice crystals [29]. It is mainly suitable for large-scale industrial production. The disadvantage is that the air freezing rate is low, resulting in large ice crystal size, low thermal conductivity of food, and poor convective heat transfer coefficient related to air. Due to the direct contact between air and meat products during the freezing process, it can also cause quality loss of meat products.

A comparison of air freezing with other advanced freezing techniques reveals significant limitations across multiple aspects of the former. For instance, relative to immersion freezing, air freezing demonstrates a slower freezing rate and lower heat transfer efficiency, leading to the formation of larger ice crystals that cause more pronounced damage to muscle fibers and meat microstructure [30]. Furthermore, in a study evaluating air freezing, liquid immersion freezing, and pressure shift freezing of Channa Argus, air freezing at −20 °C required the longest freezing time and resulted in the poorest quality after freezing [28].

In summary, although air freezing is a prevalent industrial technique, its characteristically slow freezing rate promotes the formation of large, extracellular ice crystals. This microstructural alteration inflicts significant mechanical damage to the muscle tissue, which manifests as a marked deterioration in key quality attributes, including reduced tenderness, discoloration, and accelerated lipid oxidation [31].

#### 3.1.2. Contact Freezing

Contact freezing facilitates efficient heat removal via direct conduction between the food and a refrigerated medium (e.g., plates, cryogenic liquids). This method achieves a much higher heat transfer coefficient than air convection, leading to rapid freezing, reduced dehydration, and improved color preservation [32].

Contact freezing, which often employs metal plates for their superior thermal conductivity, significantly enhances freezing rates. However, this method can be somewhat particular: it performs optimally with well-shaped, flat food products and requires complete packaging to ensure efficient heat transfer. Imperfect contact not only diminishes freezing efficiency but also raises concerns of potential contamination or physical damage to delicate food tissues. Yuksel Sarıoğlu and Dirim [33] found that compared to other freezing methods, the protein denaturation temperature of metal plate-assisted frozen samples was usually higher than that of the control group and ultrasound-assisted frozen samples, which is consistent with the exploration of the effects of different freezing methods on beef quality.

#### 3.1.3. Liquid Immersion Freezing

Liquid immersion freezing (LIF) achieves rapid cooling by direct contact between the food and a liquid refrigerant. LIF has garnered significant attention due to its relatively low cost and excellent heat transfer capability. It has been reported that the heat transfer coefficient of the liquid medium can reach 100–140 W/m^2^·K, which is approximately 10 times higher than air (15–17 W/m^2^·K) [34]. This fundamental advantage enables vastly faster freezing rates and shorter processing times. Common refrigerants include ethanol, glycerol, and propylene glycol. However, to mitigate the risk of solvent contamination and further enhance performance, auxiliary technologies such as high pressure, ultrasound, or magnetic fields are increasingly being integrated with immersion freezing [35].

Gan, et al. [36] investigated the effect of immersion freezing on the water holding capacity (WHC) of pork by applying different magnetic field strengths, and compared the freezing rates, myofibrillar protein content, moisture distribution, and other indicators between air freezing and single immersion freezing. It was verified that magnetic field-assisted immersion freezing can effectively improve the quality of pork.

Collectively, the evidence indicates that while immersion freezing achieves rapid freezing rates due to its high heat transfer coefficient, the technology is constrained by challenges related to processing uniformity and operational costs [37].

#### 3.1.4. Cryogenic Freezing

Cryogenic freezing mainly uses refrigerants with extremely low boiling points to directly or indirectly come into contact with food, taking away a large amount of heat through intense boiling and evaporation. Cryogenic freezing is a rapid freezing technology that can effectively improve the freezing rate by using low-temperature liquids [38]. Compared with other traditional freezing methods, cryogenic freezing can effectively reduce the risk of forming large ice crystals. Relevant research reports show that cryogenic freezing has a significant effect on shortening the freezing time [39]. Cryogenic freezing also offers significant advantages for preserving food quality during frozen storage, primarily reflected in better color retention, reduced lipid oxidation, decreased thawing loss, and enhanced microbial safety [40,41,42]. It has been reported that the cryogenic agents used in this process rapidly form an icy glaze on the food surface, which helps to minimize moisture loss [43].

However, it was observed that excessively high cooling rates can cause rapid sublimation of surface moisture, potentially leading to freeze burn and deterioration of meat quality [44]. Similarly, significant temperature gradients may be induced on the meat surface when using liquid nitrogen as a cryogenic agent, generating internal stresses that damage structural integrity [45].

Furthermore, liquid nitrogen is a prohibitively costly refrigerant that cannot be recovered after evaporation, rendering cryogenic freezing a method that poses significant technological and economic constraints. To advance conventional cryogenic techniques, future work should prioritize the development of more economical refrigerants and the optimization of freezing process parameters. The comparison of the four traditional freezing methods is shown in Table 2.

### 3.2. Evaluation of Traditional Thawing Technology

#### 3.2.1. Air Thawing

Air thawing, a conventional method for meat products, relies on natural convection and conduction between the ambient air and the product surface. The thawing rate is highly dependent on air temperature, with slower thawing occurring between 0 and 4 °C and faster thawing at 20–25 °C [46]. However, the process is inherently constrained by the low thermal conductivity and specific heat capacity of air. To improve efficiency, introducing water vapor to increase relative humidity can enhance heat transfer performance and accelerate thawing while reducing surface moisture evaporation [47].

#### 3.2.2. Water Thawing

Water thawing utilizes liquid water as a heat transfer medium, exploiting conduction and convection to melt internal ice crystals in frozen products. The high specific heat capacity and thermal conductivity of water enable efficient phase transition via direct contact with the product surface. Based on hydrodynamic conditions, this method is classified into two modes: still water thawing and flowing-water thawing [10].

Still-water thawing depends on natural convection, with thawing kinetics governed by water temperature, product load, and water volume. Although operationally simple and scalable for batch processing, this approach is characterized by prolonged thawing durations, which increase the risk of microbial proliferation. In contrast, flowing-water thawing enhances thermal exchange through forced convection, with thawing rates determined by water temperature and flow velocity. This configuration reduces processing time and restricts microbial growth, thereby better preserving post-thaw quality. However, it may also promote the leaching of water-soluble nutrients [48].

Comparative studies further illustrate these limitations. In work on filets, Li, et al. [49] observed that water immersion thawing (WIT) following rapid freezing induced marked dehydration and structural damage to muscle tissue. Moreover, WIT required approximately 30 min to thaw the meat completely—more than twice the time needed for ultrasound-assisted thawing (UAT)—underscoring the suboptimal kinetics of conventional water-based thawing.

#### 3.2.3. Vacuum Thawing

Vacuum thawing technology is a technique that utilizes the latent heat of phase change boiling of water in a vacuum state under low pressure to produce a large amount of low-temperature water vapor, which condenses on the surface of frozen materials to release heat, thereby achieving the thawing effect [50]. The core principle is to reduce environmental pressure and evaporate the internal moisture of frozen products at low temperatures (0–10 °C), using the heat absorption effect of evaporation to accelerate the melting of ice crystals. Its advantages include low juice loss rate after thawing, effective inhibition of microbial growth under vacuum, avoidance of protein denaturation and lipid oxidation, and effective preservation of meat quality. Chen, et al. [51] took frozen pork as the research object, proposed a new vacuum thawing method based on the principle of vacuum sublimation rehydration thawing (VSRT), and built a vacuum sublimation rehydration thawing testbed based on this principle.

By comparing the thawing rate, texture, thawing loss, and energy consumption of natural air thawing (NAT), it was verified that the overall thawing rate and thawing loss parameters of VSRT were significantly lower than those of natural air thawing (NAT), proving the superiority of vacuum thawing in energy consumption and thawing rate.

## 4. Exploration of New Freezing and Thawing Technology

### 4.1. Evaluation of New Freezing Technology

#### 4.1.1. High-Pressure Freezing

**Figure 2:**
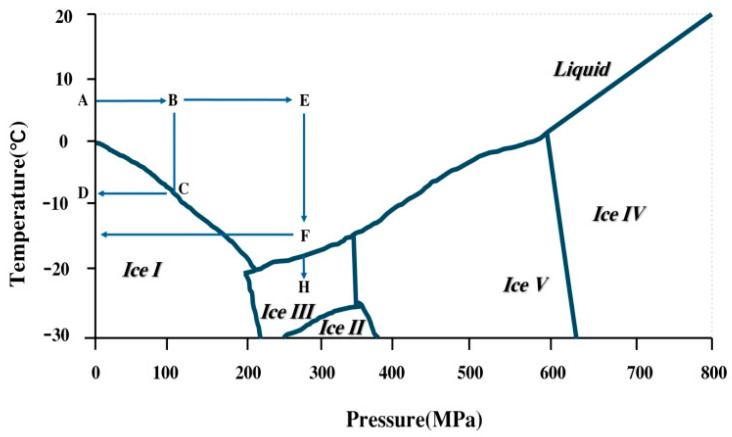
Solid–liquid equilibrium phase diagram of water under high pressure(ABCD: pressure-assisted freezing, DCBA: pressure-assisted thawing, ABEFG: pressure shift freezing, GFEBA: pressure-induced thawing).

High-pressure freezing technology (HPF) refers to the application of a high hydrostatic pressure field of 200–600 MPa during the conventional freezing process [52]. Its core lies in the influence of high pressure on the water phase diagram [53]. As shown in Figure 2, under a high-pressure environment, the freezing point of water decreases, that is, the degree of supercooling increases. Specifically, a high pressure lowers the freezing point of water, thereby increasing the degree of supercooling and modifying the temperature range for ice crystallization. This thermodynamic effect promotes the rapid and uniform nucleation of numerous fine ice crystals while effectively suppressing their growth and recrystallization during freezing. As a result, HPF significantly mitigates the microstructural damage to meat tissues caused by large ice crystals in conventional freezing [54].

Depending on the phase transition pathway, HPF can be categorized into three distinct modes [55]. High-Pressure-Assisted Freezing (HPAF): Phase transition occurs under constant pressure. High-Pressure Shift Freezing (HPSF): Phase transition is triggered by pressure release, utilizing the instantaneous and uniform supercooling generated across the entire sample volume to form numerous small ice crystals. High-Pressure-Induced Freezing (HPIF): Phase transition is initiated by pressure increase and continues under constant pressure.

The superior microstructure preservation achieved by HPF, particularly HPSF, directly translates to enhanced product quality. Studies have demonstrated significant improvements in water-holding capacity, reduced drip loss, better color preservation, and minimized protein denaturation compared to conventional methods. For instance, Martino, et al. [56] compared the quality and structure of pork frozen by high-pressure shift freezing (200 MPa, −20 °C), air-blast freezing, and liquid nitrogen freezing. Their results indicated that HPSF produced the smallest ice crystals and induced the least damage from internal stress, underscoring its advantage in maintaining product integrity. According to Table 3, it reflects that high-pressure freezing technology is widely used in meat products.

The future advancement and industrial implementation of high-pressure freezing (HPF) critically depend on achieving precise, predictable control over its complex multi-physics process. The core prospect lies in the synergistic integration of artificial intelligence (AI) with foundational mechanistic models, specifically targeting two intertwined modeling challenges: accurate heat and mass transfer simulation under pressure and predictive kinetics of quality evolution.

Current models for high-pressure heat transfer are hindered by difficulties in defining pressure-dependent thermophysical properties of biomaterials and simulating transient phase-change events during pressure shifts. Concurrently, describing the long-term quality degradation of HPF-treated meat requires sophisticated kinetic models that link initial microstructural states from freezing to subsequent biochemical pathways in storage [57].

AI-driven optimization algorithms can utilize these integrated digital twins—combining the thermal model and the quality kinetic model—to perform inverse design. They can identify the optimal set of process parameters (pressure level, cooling rate, and holding time) required to achieve a target outcome [55].

Therefore, the path forward for HPF is the development of intelligent, model-based control systems. By fusing AI’s pattern-finding power with the mechanistic understanding from thermal and kinetic models, these systems will enable the precise, tailored, and reliable parameter control necessary to transition HPF from a promising laboratory technique to a robust, scalable, and economically viable technology for high-quality meat processing.

**Table 3:** Application of high-pressure-assisted freezing technology in meat products.

Meat Product	Pressure Parameters	Treatment Conditions	Effect	Reference
Frozen pork	200 MPa	After cooling to −20 °C at a pressure of 200 MPa	Meat products under high-pressure freezing form small and uniform ice crystals	[56]
Frozen shrimp meat	550 MPa	HPP processing at 50 MPa for 10 min	Accelerate water migration in shrimp and reduce mechanical damage to muscle fibers	[58]
Frozen perch	200 MPa	Increase the pressure to 200 MPa at a rate of 3 MPa/s	The achieved ice crystals are smaller, improving cell integrity	[59]
Frozen beef	650 MPa	Apply pressure at room temperature (20 °C) (650 MPa/10 min)	Beef significantly increases its expressible water content and effectively maintains its color under high-pressure treatment	[60]

#### 4.1.2. Ultrasound-Assisted Freezing

**Figure 3:**
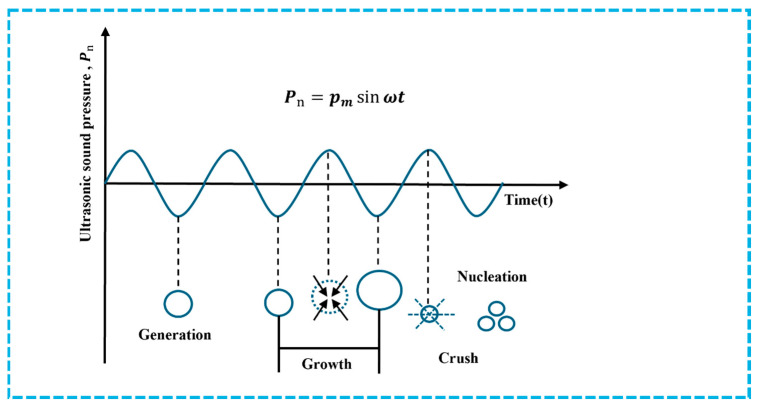
Cavitation effect of ultrasound.

Ultrasonic-assisted freezing utilizes the cavitation effect and mechanical vibration generated by ultrasound propagation in meat products to promote ice crystal nucleation, inhibit recrystallization, improve the freezing rate of frozen meat products [61], and increase the number while decreasing the size of ice crystals. cavitation effect refers to the generation and instantaneous collapse of micro-sized bubbles in liquid by ultrasound, releasing shock waves and local high temperatures and high pressures. The specific principle of ultrasound cavitation effect is shown in Figure 3. Cavitation bubbles can effectively reduce the supercooling temperature required for ice crystal nucleation, thereby promoting uniform nucleation [62]. Appropriate ultrasound-assisted freezing can effectively improve sample quality and reduce ice crystal damage to meat tissue during the freezing process. As shown in Table 4, ultrasound-assisted freezing technology is also widely used in meat products.

Chen, et al. [63] studied the effect of ultrasound-assisted immersion freezing with a frequency of 45 kHz and a power of 320 W on the storage quality of sea bass. By freezing sea bass slices for storage (0, 2, 4, 6, 8, and 10 weeks), the ice crystal morphology was found to be significantly smaller and more uniform compared to the frozen group (QF), indicating that ultrasound can effectively increase ice crystal nucleation and reduce ice crystal size. Li, et al. [64] also investigated the effects of ultrasound-assisted immersion freezing (UIF) at different power levels (0, 200, 400, and 600 W) on the quality and flavor of stewed beef. By analyzing indicators such as juice loss, fat oxidation TBARS value, and beef tenderness after freezing, it was confirmed that a 400 W UIF treatment could significantly reduce juice loss, fat oxidation degree, and tenderness in braised beef. Among them, steaming damage decreased from 49.04% to 39.74%, and TBARS value decreased from 0.32 mg/kg to 0.20 mg/kg. The results showed that ultrasound-assisted freezing could significantly improve the flavor and quality of processed beef.

Despite its efficacy in refining ice crystal structure and enhancing meat quality at the laboratory scale, the industrial adoption of ultrasound-assisted freezing (UAF) faces significant challenges rooted in its operational mechanisms [65]. The beneficial cavitation and nucleation effects are often accompanied by undesirable thermal and mechanical impacts, which can cause protein oxidation, cellular damage, and non-uniform texture in heterogeneous food matrices under suboptimal conditions [66,67]. This issue is compounded by a lack of standardized protocols across studies, where variations in ultrasonic parameters and sample properties hinder the development of reproducible industrial processes.

Currently, the application of ultrasound-assisted freezing (UAF) in food processing remains predominantly at the laboratory research and small-batch pilot scale, with a notable absence of standardized, industrial-scale equipment for continuous production. Its translation to widespread industrial deployment is constrained by significant technical challenges—particularly in achieving uniform and controlled acoustic effects at scale—and associated economic feasibility concerns for large-volume operations. Future progress toward reliable and cost-effective industrialization is contingent upon a deeper mechanistic understanding of ultrasound-enhanced heat and mass transfer, coupled with technological advances in real-time process control and the establishment of unified processing standards.

**Table 4:** Application of ultrasound-assisted freezing technology in meat products.

Meat Product	Ultrasound Parameters	Treatment Conditions	Effect	Reference
Carp	30 kHz, 175 W	9 min ultrasound (30 s on/off cycle) prior to freezing	Significantly reduced thawing loss	[63]
Chicken breast	40 kHz, 50 W	Intermittent ultrasound with variable net treatment times	Shortened freezing time	[68]
Pork	0–300 W	Ethanol-fluoride coolant (95:5) bath system	Minimum thawing loss at 180 W	[17]
Beef myofibrillar protein	200–600 W	Compared with air/immersion freezing (AF/IF)	Delayed structural deterioration and thermal stability loss	[69]

#### 4.1.3. Electric Field-Assisted Freezing

Electric field-assisted freezing technology changes the dipole moment of water molecules by applying an external electric field [70]. Based on the directional polarization effect of the electric field on polar water molecules, the free energy is reduced, thereby changing the arrangement and motion of water molecules, accelerating ice crystal formation, and inhibiting the growth of large ice crystals [71]. Research has shown that an electric field of appropriate intensity and frequency can control the size and shape of ice crystals in pork, lamb, and beef, and improve freezing rates [72]. Table 5 reflects the current application of electric field-assisted technology in the field of meat products.

Xanthakis, et al. [73] studied the effect of electrostatic field (SEF) on the freezing of pork (pork tenderloin). By controlling the intensity of the SEF, it was observed that the average equivalent circle diameter of ice crystals significantly decreased from 32.79 ± 4.04 μm in the initial control group to 14.55 ± 8.20 μm in the sample frozen under the maximum electric field. Dalvi-Isfahan, et al. [74] investigated the quality effect of lamb meat under electrostatic field (ESF) and found that ESF freezing maintains the hardness and microstructure of lamb meat, reduces water loss after thawing, and efficiently ensures the quality of frozen lamb meat.

In addition to the application of electrostatic fields in the field of meat freezing, alternating electric field (AEF) technology is also a modern freezing assistance technology that can be used to improve meat quality. According to research reports, AEF can prolong the nucleation time of ice crystals, increase the undercooling of meat products, and thus reduce the size of ice crystals formed during the freezing process. Wu, et al. [75] studied the effect of AEF on the quality of frozen beef and verified that AEF can reduce cleaning loss, cooking loss, and increase meat tenderness to maintain meat quality; Lin, et al. [76] investigated the changes in beef freezing parameters and temperature distribution induced by AEF, and designed a special system to dynamically monitor the parameters. The results showed that freezing assisted by alternating current (AEF) can significantly reduce the myofibrillar fragmentation index (MFI) of beef, and the equivalent ice areas of surface, middle, and internal samples treated with AEF were 8.16, 14.40, and 20.56%, respectively, which were significantly lower than those of traditional freezing (15.87, 24.60, and 28.8%). The analysis indicates that AEF-assisted freezing effectively modulates the characteristic parameters of the freezing curve and mitigates freezing-induced damage.

Despite compelling laboratory evidence of its efficacy in ice crystal control, the industrial adoption of electric field-assisted freezing (EFAF) has been impeded by formidable practical barriers. The core challenge lies in translating its theoretical mechanism into robust, large-scale engineering. Its contents mainly include the modification of industrial equipment, the adjustment of the uniformity of parallel plate electrodes, and the improvement of energy efficiency [77].

These issues, coupled with the need for specialized equipment, result in high capital and operational costs, rendering its current industrial readiness low. Consequently, EFAF remains largely confined to academic and prototype-scale research. In the future, electric field-assisted freezing also needs to be developed cooperatively. Combined with other physical fields and machine learning algorithms, it is necessary to build a robust multi-parameter optimization model and dynamically adjust the electric field parameters through the algorithm [78] so as to adapt to different materials and different characteristics of food freezing and improve its scalability.

**Table 5:** Application of electric field-assisted freezing in meat products.

Meat Product	Parameters	Conclusion	Reference
Pork tenderloin	Voltage: 0–12 kV; Power: 8.9–11.6 W	Static electric field (SEF) can significantly improve the microstructure damage of meat products	[73]
Lamb Filet	Voltage: 0, 4, 8, and 12 kV	Static electric field maintains quality during the lamb-freezing process	[74]
Beef	Voltage: 2200 V, AC220 V; Current: 0.2 mA; Frequency: 50/60 Hz	The application of alternating electric field (AEF) can significantly improve the quality of beef	[75]
Beef muscle	Voltage: 220 V; Frequency: 50/60 Hz	AEF-assisted freezing adjusts the freezing parameters of beef and reduces freezing damage	[76]
Yak meat	Field strength: 1800 V/m; Frequency: 300 Hz	AEF avoids ice-induced freezing damage and maintains the integrity of meat products	[79]

### 4.2. Evaluation of New Thawing Technology

#### 4.2.1. Microwave Thawing

Microwaves are electromagnetic waves with frequencies ranging from 300 MHz to 300 GHz [80]. The essence of microwave thawing is to use high-frequency electromagnetic waves to penetrate the interior of meat products, causing polar molecules (such as water molecules) in the meat products to rapidly vibrate and generate heat, achieving rapid thawing. Compared with traditional natural thawing and hydrolysis thawing, microwave thawing has the characteristics of shorter thawing time, uniform heating inside and outside, and less meat loss [81]. Microwave thawing can also improve the flavor and quality of meat products to a lesser extent [82]. Data shows that microwave thawing can improve the water retention of muscle foods and inhibit the growth of microorganisms [83].

Hu, et al. [84] studied the effects of infrared and microwave heating on the quality of frozen pork tenderloin. By measuring indicators such as moisture status, texture, pH value, and TBARS value, it was found that, when using 1.92 W/g wet pork with a microwave intensity of 50 W, the minimum loss was only about 1.7%, confirming that microwave thawing can effectively reduce the thawing loss of pork tenderloin. James, et al. [85] found that microwave thawing is the fastest and most effective method to maintain pork quality, but thawing the meat can cause local overheating, resulting in poor meat quality.

Rakesh, et al. [86] developed a microwave thawing device where hot air was generated by heating elements and circulated through a rotating fan located at the back of the oven. Thawing was achieved using a combined microwave and hot air system, with microwaves introduced through a top waveguide.

The above research indicates that the advantage of microwave thawing lies in its fast thawing rate and low thawing loss rate. However, this technology has disadvantages such as high equipment cost, uneven thawing, and local heating. Therefore, special attention should be paid to optimizing the parameters of microwave thawing to minimize potential problems. In summary, microwave thawing is still an effective measure for thawing meat products.

Future progress relies on integrating microwave energy within hybrid multi-physical field systems to achieve synergistic, uniform thawing. Ultimately, the implementation of AI-driven, model-based control systems is essential to dynamically optimize these combined parameters in real-time, ensuring precise, quality-focused, and scalable industrial applications.

#### 4.2.2. Ohmic Heating Thawing

Ohmic thawing is an advanced technology that utilizes frozen meat itself as a resistive element, where the application of a low-frequency alternating current induces volumetric Joule heating within the product, enabling rapid and uniform thawing [87]. This method is characterized by uniform heating, high energy utilization efficiency, and significantly shortened thawing times [88]. Research has demonstrated that the voltage gradient applied during Ohmic thawing plays a critical role in influencing the textural properties of thawed meat. For instance, Icier, et al. [89] observed that the voltage gradient significantly affects the final texture of beef chunks thawed via this method. Further investigation by Cevik and Icier into the effects of different voltage gradients (10, 13, and 16 V/cm) on the electrical conductivity of frozen beef revealed that the effective conductivity follows a polynomial model, providing valuable insights for the optimization of industrial Ohmic thawing systems for meat products [88].

Meat products thawed using Ohmic heating exhibit excellent retention of color, flavor, and nutritional quality. Tian, et al. [90] developed a device that uses Ohmic heating to thaw meat products. In a systematic study, Bozkurt and Içier [91] applied varying voltage gradients (10, 20, and 30 V/cm) to analyze changes in thawing time, drip loss, and color of frozen beef chunks. Their results confirmed that increasing the voltage gradient significantly reduces thawing time while maintaining stable thawing loss, indicating that Ohmic thawing can achieve high efficiency without compromising product quality. To further enhance thawing performance, Min, et al. [92] developed a pressure-assisted Ohmic thawing (POT) system, which thaws frozen beef using Ohmic heating (200 V/cm) under high pressure (40 MPa). Comparative analysis of conventional thawing, standalone Ohmic thawing, and pressure-assisted thawing demonstrated that the POT combination resulted in the shortest thawing time (0.8 min). Moreover, POT effectively minimized alterations in shear force, thereby better preserving the meat’s original texture.

In summary, Ohmic thawing represents a promising alternative technology for the meat industry. Its rapid and efficient thawing mechanism offers significant potential for industrial application. Compared to traditional thawing methods, Ohmic thawing provides more precise process control and reliably ensures the preservation of quality in frozen meat products.

#### 4.2.3. Radio Frequency Thawing

Traditional thawing mainly involves transferring heat from the outside to the inside of frozen meat products through external heat sources and media. Radio frequency (RF) is essentially a form of electromagnetic wave energy. RF thawing uses a high-frequency electric field to repeatedly polarize and vibrate water molecules in meat products. Through the friction between polarized molecules and charged ions, their internal energy increases, generating heat. Its advantage lies in the ability to penetrate thicker meat products, achieve deep heating, fast and uniform thawing speed, and low energy consumption [93], overcoming the difficulties of traditional thawing such as long thawing time, low heat transfer efficiency, and poor food quality [8].

At present, radiofrequency thawing technology has broad applications in the thawing of large meat products [94,95]. Research mainly focuses on optimizing the distribution of radio frequency field and field strength to meet the thawing needs of different meat products. Sun et al. [95] investigated the effect of radiofrequency thawing on the quality characteristics of frozen lamb meat by applying a 6 kW, 27.12 MHz radiofrequency system. The study found that compared to air thawing, radiofrequency thawing can significantly reduce thawing time and thawing losses in the range of −18–4 °C, which has great research value in the field of meat products; Bedane et al. [94] studied frozen chicken breast meat and investigated the changes in electric field distribution and thawing uniformity with quality parameters (drip loss and texture change) in an electrode radiofrequency system. The results showed that radiofrequency thawing could achieve minimal drip loss and significantly improve texture structure.

However, the advancement of radiofrequency (RF) thawing is currently constrained by persistent challenges, including high energy costs, uneven temperature distribution and limited adaptability to products of complex geometry. The convergence of artificial intelligence (AI) and multi-physics simulation represents a pivotal direction for overcoming the inherent challenges of radiofrequency (RF) thawing. While established computational approaches—such as the COMSOL Multiphysics (V. 5.6, COMSOL AB, Stockholm, Sweden) multi-physics models used to study air-impingement-assisted RF thawing or the numerical analysis of irregularly shaped fish—provide valuable insights into temperature distribution and process parameters, they remain computationally intensive and scenario-dependent [9,96].

Future progress will depend on AI-driven frameworks embedded within multi-physics simulations. These systems will use surrogate modeling and inverse optimization—such as reinforcement learning for dynamic control—to autonomously explore the high-dimensional parameter space of electrode design, field distribution, and convective settings. This enables the co-optimization of thawing efficiency, temperature uniformity, and energy use. By integrating AI with simulation, RF thawing can evolve from a static, trial-and-error process into an adaptive, intelligently controlled technology capable of responding in real time to product variability and process disturbances, thereby improving both performance and industrial scalability.

## 5. Application of Artificial Intelligence in Freezing and Thawing

### 5.1. Prospects of Artificial Intelligence Applications

The traditional food freezing and thawing process may have problems such as long thawing time, low thawing efficiency, poor quality of meat products, and microbial contamination after thawing. Although new freezing and thawing technologies have effectively improved these problems, there are also some drawbacks. Despite its convenience, microwave thawing is often uneven. Conversely, high-voltage electrostatic field thawing, while effective, is characterized by high operating voltages that raise safety concerns.

At present, the research focus in the field of food freezing and thawing is on controlling the formation of ice crystals, mainly by accelerating the growth of small ice crystals and inhibiting the formation of large ice crystals through physical fields, antifreeze agents, temperature control, and other methods. In this regard, the application of intelligent technology can effectively establish environmental models, analyze data to predict freezing time, perform temperature control and process optimization, and further monitor the quality of frozen meat products [97].

**Figure 4:**
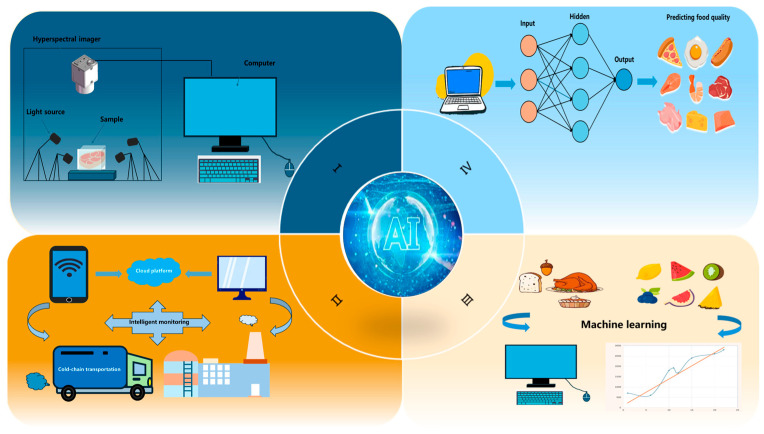
The application of AI in the food industry.

As shown in Figure 4, AI is mainly used in the field of meat freezing and thawing for numerical modeling of frozen products, establishment of prediction models for thawing parameters, and product quality monitoring of frozen products.

AI is a branch of computer science that primarily simulates human intelligent behavior through computers [98]. The current learning types of artificial intelligence include random forest (RF), decision tree (DT), support vector machine (SVM), neural network, deep learning, etc., with functions such as language recognition, image recognition, natural language processing, and expert systems. Table 6 shows us the relevant application scenarios of artificial intelligence in the food industry.

### 5.2. Numerical Modeling of Frozen Meat Products

Numerical modeling is mainly used to analyze food processes in order to better understand the complex physical mechanisms involved, evaluate food processes to ensure product quality, and design and optimize food process systems. Currently, the most widely used methods for solving fluid dynamics and heat and mass transfer problems are the finite element method, finite difference method, and finite volume method [109].

Computational simulation plays an important role in the food processing process. In a heated or cooled fluid environment, the velocity vector, streamline, temperature, pressure, and parameters of solid or liquid food can be explained more intuitively through animation [110].

Numerical modeling is currently widely used in frozen products, and related research is mainly conducted by establishing mathematical models of frozen products and using fluid dynamics simulation in computers to simulate the state changes and heat and mass transfer processes of the freezing process. Shan and Heldman [111] established a transient heat transfer model using the finite element method (FEM) to generate the tempera-ture distribution history in the studied frozen pork products, providing faster calculations and more direct data post-processing. In order to address the complexity of numerical solutions for heat transfer problems in high-moisture foods. Shan, et al. [112] determined the heat transfer model and developed different methods based on curve fitting, the use of S-shaped functions, data interpolation and evaluated their performance of these methods in numerical simulations of food freezing and thawing processes.

In the numerical simulation of frozen food, Computational Fluid Dynamics (CFD) is widely used in the field of freezing, refrigeration, and preservation to explore the modeling of heat and mass transfer in food during the process. Hu and Sun [113] simulated the heat and mass transfer of ham in blast cooling using CFD to predict the cooling rate and weight loss during the cooling process.

Current research predominantly centers on two areas: establishing mathematical models for frozen environment simulation, and the detailed physical modeling of product attributes—including geometry, texture, and color evolution—during coupled heat and mass transfer processes.

Ouyang, et al. [114] has developed a model for thawing pork using ultrasound through numerical simulation. The RMSE value predicted by the model for thawing temperature is 0.09–0.32, which is important for exploring the heat transfer process of ultrasound thermal effect. Cevik and Icier [115] successfully simulated the temperature distribution of Ohmic thawing of meat samples using finite volume FV with the help of ANSYS software (Academic Version CFD module). Pham [116] explored the modeling of heat and mass transfer in frozen food and pointed out that although CFD is currently popular for calculating heat transfer coefficients, practical models are still lacking in areas such as circulation flow and natural convection.

Studies have demonstrated that numerical modeling has become a cornerstone in understanding and optimizing food processing, particularly for freezing and thawing operations. By employing methods such as the finite element, finite difference, and finite volume approaches, simulations enable detailed analysis of complex multi-physics phenomena—including coupled heat and mass transfer—in frozen products. Current research is increasingly focused on integrating these computational techniques with advanced physical thawing methods, such as radiofrequency (RF) and ultrasound-assisted processes. For instance, numerical models are being developed to simulate RF field distribution for improving heating uniformity, and to predict temperature profiles with high accuracy [9,96]. This synergy between targeted thawing technologies and high-fidelity simulation offers significant advantages: it reduces reliance on costly and time-consuming empirical trials, enhances process efficiency through predictive optimization of parameters, and improves scalability by enabling the virtual design and testing of industrial-scale systems. Ultimately, the integration of numerical modeling with physical-field-based thawing represents a promising pathway toward more controlled, energy-efficient, and quality-preserving frozen food processing.

### 5.3. Quality Monitoring of Frozen Meat Products

In addition to the numerical modeling of frozen products, the application of AI in the field of food engineering also focuses on quality monitoring and nondestructive testing of food. Over decades of research in quality monitoring and non-destructive testing, most of them combine image processing and machine learning, mainly through the key parameters of quality characteristics for real-time quality monitoring of frozen food. Qiu, et al. [117] reviewed methods for identifying and detecting frozen muscle foods based on spectral technology and machine learning, including feature selection, feature extraction, and model training techniques based on ML spectral technology. Jiang et al. [58] designed a non-destructive quality determination technique for frozen food based on near-infrared (NIR) spectroscopy using machine learning. By establishing food models with different moisture contents, different algorithms such as principal component regression (PCR), support vector machine regression (SVR), partial least squares regression (PLSR), and backpropagation artificial neural networks (BP-ANN) were compared for their predictive performance. Consequently, the BP-ANN model demonstrated superior overall performance compared to other models.

Parastar, et al. [118] developed a fast and non-destructive detection method for identifying the authenticity of chicken meat by combining portable handheld near-infrared (NIR) spectroscopy with machine learning. The accuracy of a single scan can reach over 95%.

In addition to near-infrared spectroscopy technology, low-field nuclear magnetic resonance (LF-NMR) is also widely used in the monitoring of food. LF-NMR can quickly detect water states (bound, fixed, and free water) by analyzing proton relaxation time (T) [119]. With this characteristic, Jiang, et al. [120] applied LF-NMR to non-destructive testing of frozen food and constructed a deep learning detection model for frozen food based on low-field nuclear magnetic resonance, demonstrating the superiority of the BP-ANN model in predicting dripping and texture.

The above detection technologies can be popularized, but relatively speaking, quality detection in frozen samples still lack targeted practical application scenarios. Consequently, a deeper understanding of the fundamental mechanisms behind these quality detection techniques is essential for advancing the field. These AI-enhanced methods offer notable advantages in speed and repeatability compared to traditional analyses. However, their scalability to industrial settings remains constrained by factors such as high instrument costs, the need for extensive and product-specific calibration datasets, and challenges in real-time integration into fast-moving processing lines. Furthermore, many studies remain at the laboratory scale, with limited validation under real-world freezing conditions and variable product geometries. Future efforts should therefore focus on developing more robust, generalizable models and cost-effective sensor systems to bridge the gap between proof-of-concept research and widespread industrial adoption in frozen food quality assurance.

### 5.4. Prediction of Food Thawing Parameters

In the field of prediction of thawing parameters of frozen products, most of the articles focus on the establishment and prediction of key parameters such as time and rate in the thawing process through neural networks and heat and mass transfer direction.

In terms of food freezing and thawing, the detection of freezing and thawing time and frozen product quality is currently a field where artificial intelligence is widely applied. Boillereaux et al. [121] used real-time neural network learning to estimate the thermal characteristics during the thawing process and predict the duration of food thawing; Goñi, et al. [122] developed a neural network-based model for predicting food freezing time, which uses input variables such as product thickness, width, convective heat transfer coefficient, frozen product thermal conductivity, specific heat, and moisture content to achieve simple and fast calculations. Different from traditional thawing methods, Cheng, et al. [123] developed a prediction model that accurately identifies the thawing endpoint using ultrasound signals and ultrasound velocity. Its $R^{2}$ value exceeds 0.9.

Cheng et al. [123] constructed a prediction model for the moisture content of frozen pork using hyperspectral imaging combined with electronic nose technology, which mainly includes sample preparation, hyperspectral image acquisition, data acquisition, spectral image, and PLSR modeling analysis.

The application of artificial intelligence, particularly neural networks, in predicting critical thawing parameters—such as time, rate, and endpoint—demonstrates significant advantages in terms of prediction accuracy, computational efficiency, and reduced dependence on experimental validation. Emerging approaches integrating non-destructive techniques like ultrasound and hyperspectral imaging with machine learning further enable real-time, in-process monitoring. However, the scalability and generalizability of these models remain constrained, as most are developed and validated under conventional thawing conditions and are often product-specific. A more pressing limitation lies in the insufficient adaptation of predictive frameworks to advanced thawing technologies—such as radiofrequency, ultrasound-assisted, and high-pressure processing—where complex multi-physics interactions are not adequately represented in existing data-driven models.

Future research should prioritize the development of hybrid modeling frameworks that integrate physics-based simulations with machine learning to better capture synergistic multi-parameter effects and support the scalable, adaptive optimization of next-generation thawing systems.

## 6. Conclusions

This review synthesizes the state of the art in meat freezing and thawing, charting a clear trajectory from foundational principles through advanced multi-physical field technologies to the transformative integration of artificial intelligence. While conventional methods remain economically viable, they are fundamentally limited by inefficient heat transfer, uncontrolled ice crystal growth, and poor adaptability, leading to inevitable quality deterioration. In contrast, emerging multi-physics technologies—including high-pressure, ultrasound-assisted, and electric field-coupled processes—demonstrate superior capabilities for regulating ice nucleation, growth, and recrystallization. By leveraging specific physical mechanisms (e.g., pressure-mediated phase diagram shifts, cavitation-induced nucleation, and dipole alignment), these approaches significantly mitigate microstructural damage, thereby better preserving key quality attributes such as water-holding capacity, texture, and oxidative stability.

The integration of AI represents a paradigm shift, moving the field from empirical optimization toward intelligent, model-driven design. AI applications, particularly in multi-physics numerical simulation, non-destructive quality monitoring, and predictive parameter modeling, offer powerful tools to overcome longstanding challenges of non-uniformity, inefficiency, and scalability. The synergy of physics-based models with machine learning enables virtual prototyping, real-time adaptive control, and the autonomous optimization of complex, multi-variable processes—most notably in advanced thawing systems like radiofrequency and ohmic heating.

However, the path to widespread industrial adoption is not without obstacles. High capital costs, energy consumption, and the need for robust, generalized AI models that can adapt to diverse meat products and processing scales remain significant barriers. Furthermore, a deeper mechanistic understanding of multi-physical field interactions and their synergistic effects on ice crystal dynamics is essential for future innovation.

Therefore, future research should prioritize the development of cost-effective, scalable intelligent systems that seamlessly merge multi-physics mechanisms with AI-driven control. Advancing hybrid modeling frameworks and establishing standardized protocols will be critical to realizing a new generation of sustainable, efficient, and quality-centric meat freezing and thawing processes for the global supply chain.

## Figures and Tables

**Table 1:** Impact of recrystallization on meat quality.

Meat Product	Conditions	Impact Parameters	Specific Effects	Reference
Frozen beef	4 °C refrigeration	Crystal size	Disrupt muscle fiber membranes increase drip loss	[23]
Frozen lamb tenderloin	1.5 °C aging	Deterioration index	Ice crystal coarsening muscle fiber fracture post-thaw hardening	[24]
Frozen pork tenderloin	−12 °C rapid freezing	Structural stability	Induce muscle fiber damage Compromise oxidative stability	[25]
Frozen steak	−12 °C slow freezing	Sensory quality	Harden texture and reduce tenderness	[26]
Frozen lamb loin	Post-rapid freezing thawing	Cell damage	Trigger intracellular freezing injury	[27]

**Table 2:** Comparative analysis of conventional freezing technologies.

Technology	Freezing Principle	Advantages	Disadvantages	Typical Applications
Air Freezing	Convective heat transfer with forced cold air	Cost-effectiveness	Slow freezing rate	Bulk meats, baked goods
Contact Freezing	Conductive heat transfer through refrigerated plates.	High efficiency	Geometric constraints	Block-shaped product
Immersion Freezing	Direct heat exchange via food-grade coolant immersion.	Rapid freezing	Potential contamination	QF seafood, berries
Cryogenic Freezing	Ultra-rapid heat absorption by liquefied gas	Superior quality	High operational cost	High-value delicate foods

**Table 6:** Application of artificial intelligence in the field of meat processing.

Learning Type	Core Functions	Application Examples	References
Decision tree	Classification Regression	1. Using a decision tree based on feed factors and carcass characteristics to predict beef tenderness 2. Using decision trees and hyperspectral imaging to classify beef marble patterns	[99,100]
Random forest	Classification; Outlier detection	1. Revealing changes in characteristic compounds in refrigerated pork through a random forest regression model 2. Using the RForest algorithm to detect beef freshness through bioimpedance spectroscopy	[101,102]
Support vector machine	Binary classification multi-classification	1. Using computer vision and support vector machines for color grading of bovine fat 2. Evaluate meat freshness through support vector machines	[103,104]
Neural network	Nonlinear function fitting	1. Identification of microbial populations in spoiled meat using electronic nose coupled with fuzzy wavelet network 2. Using convolutional neural network to detect chicken meat types through image feature extraction	[105,106]
Deep learning	Complex pattern learning; Multimodal fusion	1. Applying a new deep 3D convolutional neural network model for classifying meat species in hyperspectral images 2. Using deep learning neural network models to classify and recognize beef cuts	[107,108]

## Data Availability

The original contributions presented in the study are included in the article, and further inquiries can be directed to the corresponding author.
